# Sepsis Attributed to Bacterial Contamination of Platelets Associated with a Potential Common Source — Multiple States, 2018

**DOI:** 10.15585/mmwr.mm6823a2

**Published:** 2019-06-14

**Authors:** Sydney A. Jones, Jefferson M. Jones, Vivian Leung, Allyn K. Nakashima, Kelly F. Oakeson, Amanda R. Smith, Robert Hunter, Janice J. Kim, Melissa Cumming, Eileen McHale, Pampee P. Young, Joy L. Fridey, Walter E. Kelley, Susan L. Stramer, Stephen J. Wagner, F. Bernadette West, Ross Herron, Edward Snyder, Jeanne E. Hendrickson, David R. Peaper, Adi V. Gundlapalli, Charles Langelier, Steve Miller, Ashok Nambiar, Morvarid Moayeri, Jack Kamm, Heather Moulton-Meissner, Pallavi Annambhotla, Paige Gable, Gillian A. McAllister, Erin Breaker, Erisa Sula, Alison Laufer Halpin, Sridhar V. Basavaraju

**Affiliations:** ^1^Epidemic Intelligence Service, CDC; ^2^Connecticut Department of Public Health; ^3^Division of Healthcare Quality Promotion, National Center for Emerging and Zoonotic Infectious Diseases, CDC; ^4^Utah Department of Health; ^5^Utah Public Health Laboratory, Taylorsville, Utah; ^6^California Department of Public Health; ^7^Massachusetts Department of Public Health; ^8^American Red Cross Blood Services National Headquarters, Washington, DC; ^9^American Red Cross Blood Services, Pomona, California; ^10^American Red Cross Blood Services, Salt Lake City, Utah; ^11^Scientific Affairs, American Red Cross, Gaithersburg, Maryland; ^12^Transfusion Innovation, American Red Cross, Rockville, Maryland; ^13^American Red Cross Blood Services, Farmington, Connecticut; ^14^Yale University, New Haven, Connecticut; ^15^VA Salt Lake City Health Care System, Salt Lake City, Utah; ^16^University of Utah School of Medicine, Salt Lake City; ^17^University of California, San Francisco; ^18^Chan Zuckerberg Biohub, San Francisco, California; ^19^Oak Ridge Associated Universities, Oak Ridge, Tennessee.

During May–October 2018, four patients from three states experienced sepsis after transfusion of apheresis platelets contaminated with *Acinetobacter calcoaceticus-baumannii* complex (ACBC) and *Staphylococcus saprophyticus*; one patient died. ACBC isolates from patients’ blood, transfused platelet residuals, and two environmental samples were closely related by whole genome sequencing. *S. saprophyticus* isolates from two patients’ blood, three transfused platelet residuals, and one hospital environmental sample formed two whole genome sequencing clusters. This whole genome sequencing analysis indicated a potential common source of bacterial contamination; investigation into the contamination source continues. All platelet donations were collected using apheresis cell separator machines and collection sets from the same manufacturer; two of three collection sets were from the same lot. One implicated platelet unit had been treated with pathogen-inactivation technology, and two had tested negative with a rapid bacterial detection device after negative primary culture. Because platelets are usually stored at room temperature, bacteria in contaminated platelet units can proliferate to clinically relevant levels by the time of transfusion. Clinicians should monitor for sepsis after platelet transfusions even after implementation of bacterial contamination mitigation strategies. Recognizing adverse transfusion reactions and reporting to the platelet supplier and hemovigilance systems is crucial for public health practitioners to detect and prevent sepsis associated with contaminated platelets.

## Investigation and Results

**California.** On May 4, a male patient with acute lymphoblastic leukemia (patient A) received pathogen-reduced apheresis platelets at hospital A in California (Figure). Within minutes of completing the transfusion, he briefly experienced rigors, followed 2 hours later by fever and hypotension. He was transferred to the intensive care unit for management of septic shock and recovered fully. Posttransfusion patient blood cultures (obtained 2 hours after vancomycin administration) grew only ACBC. Gram stain of the implicated platelet bag residual revealed gram-positive cocci in pairs or chains; culture of the platelet bag residual grew ACBC and *S. saprophyticus*. The implicated platelet unit was one of two platelet units manufactured from a single apheresis donation collected 5 days earlier in California. Pathogen inactivation was performed 13.5 hours after collection. Hospital A located the second platelet unit (which had not been transfused), quarantined it, and notified the blood supplier. Gram stain and culture of this platelet unit were negative. Samples obtained from the donor’s skin were culture-negative for ACBC and *S. saprophyticus.* Environmental samples obtained weeks later from the platelet collection facility and hospital A yielded no relevant organisms; however, sampled areas had been cleaned in the interim.

**FIGURE:**
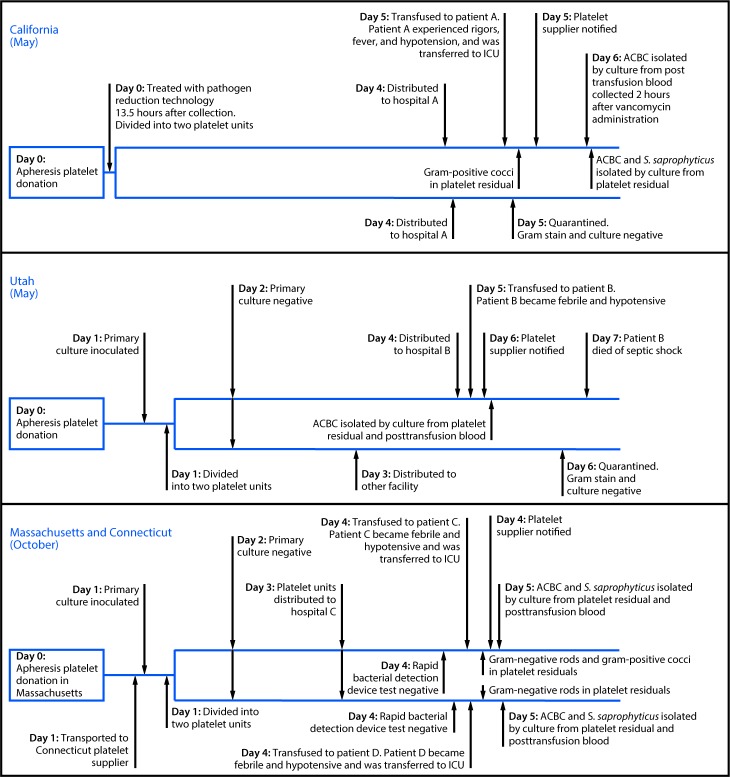
Timeline of four cases of sepsis attributed to bacterial contamination of platelets — California, Utah, Massachusetts, and Connecticut, 2018 **Abbreviations:** ACBC = *Acinetobacter calcoaceticus-baumannii* complex; ICU = intensive care unit; *S. saprophyticus* = *Staphylococcus saprophyticus.*

**Utah.** On May 10, a male patient with cirrhosis and thrombocytopenia (patient B) received a platelet transfusion at hospital B to prevent bleeding before a procedure (Figure). One hour after transfusion began, patient B complained of chills, and the transfusion was terminated. Two hours after transfusion, he became febrile, hypotensive, and tachypneic, and antibiotics were started; he died of septic shock 2 days later. ACBC was isolated by culture from platelet bag residuals and posttransfusion blood samples from the patient. The platelet supplier was notified, and a second platelet unit manufactured from the same apheresis donation, which had not been transfused, was recalled. The platelet supplier performed primary aerobic culture of the implicated donation for bacterial contamination 24 hours after collection in Utah; the primary culture remained negative after 5 days. The implicated platelet unit was transfused 5 days after collection. Samples obtained on May 24 from the donor’s urine, perianal area, and multiple skin sites screened negative for ACBC colonization. Samples obtained from platelet agitators at the platelet manufacturing facility (May 23) and hospital B (June 7) yielded ACBC isolates.

**Connecticut and Massachusetts.** On October 4, at hospital C, two male patients with acute myeloid leukemia (patients C and D) each received a platelet unit manufactured from a common apheresis donation (Figure). Within two hours of transfusion, both patients became hypotensive and febrile. Both were transferred to the intensive care unit, and both recovered. ACBC and *S. saprophyticus* were isolated by culture from posttransfusion blood samples from both patients and from both platelet bag residuals. Hospital C notified the platelet supplier. The implicated apheresis platelet donation had been collected in Massachusetts 4 days before transfusion and processed in Connecticut. Twenty-four hours after collection, the platelet supplier performed primary aerobic and anaerobic culture for bacterial contamination. Within 5 hours before transfusion, hospital C screened both platelet units with a rapid bacterial detection device; all tests were negative. No ACBC or *S. saprophyticus* isolates were identified among environmental swabs collected at platelet supplier facilities in Connecticut (November 15) and Massachusetts (November 16); *S. saprophyticus* was isolated from one platelet agitator at hospital C on November 13.

**Multistate investigation.** On July 17, notices were issued through CDC’s Epi-X and the Infectious Diseases Society of America’s Emerging Infections Network to identify additional cases of sepsis caused by *Acinetobacter* infections with onset of symptoms within 24 hours after platelet transfusion. Three cases were reported from two states (North Carolina [patients E and F] and Michigan [patient G]).

Traceback investigation revealed that the three platelet donations implicated in the California, Utah, and Connecticut septic transfusion reactions (i.e., sepsis attributed to transfusion) were from different donors. The donors in California, Utah, and Massachusetts had no known epidemiologic links to one another and no symptoms suggesting bacteremia or illness; all were indefinitely deferred. All three apheresis donations were collected in platelet additive solution using apheresis cell separator machines and collection sets from the same manufacturer; two of three collection sets were from a single lot.

CDC performed whole genome sequencing on collected ACBC and *S. saprophyticus* isolates (Table) using standard methods (*1*). ACBC organisms were isolated by culture from posttransfusion blood samples from patients A, B, C, and D; all four associated transfused platelet bag residuals; and environmental samples from hospital B and the platelet supplier in Utah. Fourteen ACBC isolates from these sources were highly related (differing by 0–32 single nucleotide polymorphisms [SNPs] across a 95.6% core genome) (Supplementary Figure 1, https://stacks.cdc.gov/view/cdc/78727) and appear to represent a novel ACBC taxon (only 90% match to *Acinetobacter seifertii* by average nucleotide identity). In contrast, ACBC isolates from cases in North Carolina and Michigan were not closely related to isolates from cases in California, Utah, and Connecticut by whole genome sequencing (differing by 13,398–14,289 SNPs across a 30.5% core genome).

**TABLE:** Bacterial contamination mitigation strategies, posttransfusion culture results, and environmental sampling results associated with four septic transfusion reaction cases — California, Utah, and Connecticut, 2018

Source	State and patient			
	California	Utah	Connecticut	
	Patient A	Patient B	Patient C*	Patient D*
**Bacterial contamination mitigation strategy**				
Pathogen-inactivation technology	Performed	Not done	Not done	Not done
Primary culture	Not done^†^	No growth	No growth	No growth
Rapid bacterial detection device	Not done	Not done	Negative	Negative
**Posttransfusion culture**				
Patient posttransfusion blood	ACBC	ACBC	ACBC and *S. saprophyticus*	ACBC and *S. saprophyticus*
Transfused platelet unit residual	ACBC and *S. saprophyticus*	ACBC	ACBC and *S. saprophyticus*	ACBC and *S. saprophyticus*
Nontransfused platelet cocomponent	Negative	Negative	None	None
**Environmental sampling**				
Hospital	Negative	ACBC	*S. saprophyticus*	*S. saprophyticus*
Platelet supplier facility	Negative	ACBC	Negative	Negative

**Abbreviations:** ACBC = *Acinetobacter calcoaceticus-baumannii* complex; *S. saprophyticus* = *Staphylococcus saprophyticus*.

* Patients C and D each received one platelet unit manufactured from a common apheresis donation.

^†^ The Food and Drug Administration does not require primary culture if the transfused platelet unit is treated with pathogen-inactivation technology.

*S. saprophyticus* was isolated by culture from posttransfusion blood samples from patients C and D; transfused platelet bag residuals from patients A, C, and D; and an environmental sample from hospital C (Table). Whole genome sequencing analysis revealed two clusters of *S. saprophyticus* isolates. One cluster consisted of *S. saprophyticus* isolates from patient C’s blood, patient C’s platelet bag residual, and an environmental swab from hospital C (differing by 0–37 SNPs across a 94.9% core genome) (Supplementary Figure 2, https://stacks.cdc.gov/view/cdc/78728); the second cluster consisted of isolates from patient D’s blood and from patient D’s and patient A’s platelet bag residuals (difference of 1–27 SNPs across a 94.9% core genome).

## Discussion

Transfusion of platelets is more likely to result in sepsis than is transfusion of other blood products; data derived from primary cultures have indicated that approximately one in every 5,000 platelet collections is contaminated with bacteria (*2*). ACBC is not frequently reported as a contaminant of platelets (*3*). ACBC consists of gram-negative bacilli that commonly occur in wet environments and are opportunistic pathogens; ACBC organisms are resistant to desiccation, persist on environmental surfaces, and avidly adhere to plastics (*4*). Conversely, coagulase-negative *Staphylococcus* spp. are among the most common bacterial contaminants of platelets (*3*,*5*). However, *S. saprophyticus* might be less likely to contaminate platelets than other coagulase-negative *Staphylococcus* spp. because it typically resides in the gastrointestinal and urinary tracts rather than on the skin (*6*).

Whole genome sequencing analysis indicated an unidentified potential common source of bacterial contamination among the four cases of septic transfusion reactions reported here. Investigation into the contamination source continues. Although skin microflora and donor bacteremia are the most frequent sources of bacterial contamination (*7*), a cluster of septic transfusion reactions attributed to contamination of blood collection bags during manufacturing or packaging was reported in 1993 (*8*).

Food and Drug Administration regulations state that blood establishments and transfusion services must assure adequate control of the risk for bacterial contamination of platelets.* Most U.S. blood suppliers fulfill this requirement by performing a primary culture of platelet donations before transfusion (*2*). Because the risk for platelet transfusion–associated sepsis has persisted despite implementation of primary cultures, additional bacterial mitigation strategies have been implemented, including pathogen-inactivation technology, rapid bacterial detection devices, and alternative culture strategies (*2*). This report underscores the possibility that sepsis resulting from bacterial contamination of platelets can occur even with application of bacterial contamination mitigation strategies.

The consequences of septic transfusion reactions are often severe morbidity or mortality. In the cluster reported here, one of four patients died, and three recovered only after receiving intensive care. Even with implementation of bacterial contamination mitigation strategies, clinicians should continue to monitor recipients for sepsis after platelet transfusions and immediately report adverse reactions to the platelet supplier and hemovigilance systems.

**Summary**

**What is already known about this topic?**

Bacterial contamination of platelets is rare (approximately one in 5,000 platelet units) but poses serious risk to platelet transfusion recipients.

**What is added by this report?**

Sepsis resulting from bacterial contamination of platelets can occur even with implementation of bacterial mitigation strategies. Whole genome sequencing indicated a potential common source of bacterial contamination among four cases of septic transfusion reactions occurring in three states.

**What are the implications for public health practice?**

Clinicians need to monitor for sepsis after platelet transfusions even after implementation of bacterial mitigation strategies and immediately report adverse reactions to platelet suppliers and hemovigilance systems.

## Footnotes

* Food and Drug Administration. Control of Bacterial Contamination of Platelets, 21 CFR Section 606.145, 2017. https://www.accessdata.fda.gov/scripts/cdrh/cfdocs/cfcfr/CFRSearch.cfm?fr=606.145.
